# The Medical Congress at Paris

**Published:** 1846-04-01

**Authors:** 


*French Medical Congress.*—In view of the approaching effort by the profession of this Country for reform and self elevation, we have thought that an account of the recent Convention in Paris may be interesting to our readers. We therefore give place to the following summary of the results of the deliberation of that body, as communicated by a Correspondent of the London Lancet, and republished in the Bulletin of Medical Science. The reader will not fail to observe some striking differences between the system of medical instruction in this country and that of France, as well as in the relations which government sustains toward the profession in the two countries. The number, unanimity and enthusiasm of the delegates assembled at the Parisian Congress is also worthy to be noted. We fervently trust that an approaching Convention, may in some degree resemble it in these particulars. To economize space, we omit some portions of the report, embracing the speeches of the President of the Congress and the Minister of Public Instruction, together with a few paragraphs which relate to matters not applicable or interesting to the profession of this country.—EDITOR BUFFALO MEDICAL JOURNAL.

*The Medical Congress at Paris.*—The importance of the Medical Congress which has just taken place in Paris, is such as to render it imperative on us to present our readers with a detailed account of its history and proceedings, and this we now purpose doing.

The medical prefession in France, although organized in a much more efficient and liberal manner than in this country, presents still many anomalies and imperfections, which have given rise to much discussion during the last few years. The necessity of extensive modification has, indeed, been so deeply and so extensively admitted, that the Minister of Public Instruction promised last session to bring before the Chamber of Deputies in 1846, a bill calculated to remedy the evils complained of.—As with ourselves, there is so much difference of opinion respecting the nature of the reforms that are required, that it appeared probable to most of those who took any interest in the question, that unless some means were previously taken to discuss the subject, and to embody in a series of propositions the results thus arrived at, the measures adopted in the parliamentary bill would most likely please no party. Under these circumstances, a few months ago the Editor of the leading medical journal, "The Gazette des Hopitaux, or French Lancet," proposed that a medical congress composed of delegates from all parts of France, should meet in Paris, prior to the opening of the Chamber of Deputies, and discuss the entire subject of medical reform. This idea was at once responded to with enthusiasm. On the 14th of last June, a meeting of the editors of the Parisian medical journals took place, the convocation of a national congress was unanimously approved of, and it was decided that publicity should be given to the plan, and that a request should be addressed to all the medical, pharmaceutical, and veterinary societies in France, to send delegates to a preliminary meeting, to be held on the 2nd of August. At this preliminary meeting, composed of delegates from the scientific societies, and of the editors of the medical journals, a committee of fifteen members, representing the three sections of medicine, was appointed and empowered to make every necessary arrangement for the organization and convocation of the congress. The powers thus granted to the committee were strictly limited to the above points.

The first step adopted by the preliminary committee was to give as extensive publicity as possible to the decision of the meeting of the 2nd of August, respecting the congress. The members of the medical profession were addressed through the political journals, which generously gave every possible assistance, and through the medical and scientific societies; whilst, on the other hand, direct communications were made to the faculties of the medical schools, and to the most eminent men in the profession. More than three thousand five hundred collective or individual adhesions were the immediate results of these measures, and the warmest sympathy was everywhere expressed. Of these, two thousand five hundred were doctors in medicine, nine hundred "pharmaciens," (chemists and druggists,) and the remainder veterinary practitioners. * * * * * * * * * * *

Before we commence analysing the proceedings of this important assembly, it is absolutely necessary that we should give a short account of the present organization of the medical profession in France. This, however, we shall do very briefly, referring to the essay now publishing on this subject, in the LANCET, by Dr. Henry Bennet for further details.

The medical profession in France, as far as medicine and surgery are concerned, constitutes part of the University of France, which is itself, under the jurisdiction of the Minster of Public Instruction. The University of France is composed of five faculties, the faculty of theology, the faculty of sciences, the faculty of arts, (letters,) the faculty of law, and the faculty of medicine. Some of these faculties are multiple. Thus there are three distinct faculties of medicine, those of Paris, Montpelier and Strasburg. The three faculties of medicine all present the same organization, with the exception that the one in Paris has a larger number of professors, and is more efficiently composed than the other two. They all examine and grant degrees. In addition, there are a number of secondary medical schools in the larger provincial towns, the certificates of which are received for part of the curriculum, but which have no power to examine or give diplomas. The degrees awarded by the faculties of medicine are those of doctor in medicine, doctor in surgery, and officer of health, (officier de sante.) The degrees of doctor in medicine and doctor in surgery are pretty much the same, the only difference being, that to obtain that of the doctor in surgery, the thesis *must* be on a surgical subject; whereas, for the degree of doctor in medicine, it may be either on a medical or on a surgical question. The degree in surgery, therefore, is scarcely ever taken, not by one in a hundred. The curriculum being precisely the same for both, and the doctors in medicine nearly all practicing surgery and midwifery, as well as medicine, indeed, every thing that comes in their way, the distinction is not recognised either by the profession or by the public. The doctors are also obliged, previously to commencing their studies, to take out two university degrees, that of bachelor of arts in the faculty of arts, and bachelor of sciences in the faculty of sciences.

The officers of health are a very inferior grade of practitioners, from whom no university degree is demanded, and whose medical education merely extends over a period of three years. They are generally ignorant men, of illiterate habits, and have no station amongst the educated classes of the community. Their degrees are partly granted by the faculties, but principally by travelling juries, formed of three professors from each faculty, who every year make a kind of circuit. They are considered the bane of the profession, and certainly by their unequal competition with the regularly educated practitioners, do much towards destroying professional profits, and degrading the profession. A very strong feeling against them exists, and there can scarcely be a doubt but that the grade will very shortly be abolished.

The chemists and druggists, (pharmaciens) constitute a very different body from what they do with us. They alone are allowed to keep and sell medicinal preparations, unless there should be none residing within a reasonable distance of a doctor or officer of health, in which case the latter is allowed to keep and prepare medicines, but for his patients only. The pharmaciens are all graduates of the schools of Pharmacy of Paris, Montpelier and Strasburg. To obtain their degree, they have to go through a three years' course of study at one of the above schools of pharmacy, which, although under the control of the university, are distinct from the faculties of medicine. By a recent decree, the degree of bachelor of arts (letters) will henceforth be demanded of all students previous to their entering at these schools, a measure which will entail upon them an eight or ten years' previous course of classical study. They are not allowed to sell anything but drugs, or to keep on their premises, or to retail, secret remedies, or any other formulæ but those which are contained in the codex, or for which they have received a prescription signed in full by a doctor or officer of health. They are thus restricted entirely, by law to the preparation of the prescriptions of medical practitioners, of which, on the other hand, they have the monopoly. The veterinary schools are not under the jurisdiction of the university, but of the Minister of Commerce. The organization of the veterinary body is far from being as complete and as satisfactory as that of the medical and pharmaceutical.

Such is the present state of the medical profession in France. Brief as is the above sketch, it will enable us to follow the Parisian congress in its interesting and important labors. * * * * *

The following is a brief synopsis of the results arrived at by the congress, the labors of which extended from the 4th to the 14th of November:—

The division of the teaching of the medical sciences between the three faculties and the preparatory schools is advantageous. (The utility of the preparatory or secondary medical schools has been much questioned of late. By this resolution, their continuance has received the sanction of the Congress, but it is also considered advisable that they should remain strictly preparatory Schools, and only be entrusted with the first period of the education of Students, all of whom ought to complete their studies in the faculties.)

The establishment of chairs of history and medical philosophy in the three faculties is advisable; also that of one on pathological anatomy in the faculty of Montpelier. (These chairs are, at present, wanting.)

The Paris Hospitals devoted to certain special diseases ought to be made the theatre of official clinical instruction. (M. Lisfranc proposed, as an amendment, that all Hospital Physicians and Surgeons should be obliged to give clinical lectures on the patients entrusted to their charge. This amendment, however, was negatived, on the plea that it would be too great an interference with individual liberty; but the wish was expressed that the medical officers of all Hospitals should be at liberty to lecture on their patients independently of any special authorization.—They are now obliged to apply for the sanction of the civil authorities of the Hospital, by whom, however, it is never refused.)

The number of faculties now existing is sufficient, being neither too considerable, nor too small. (It had been proposed by some to abolish the faculties of Montpelier and of Strasburg, transforming them into secondary Schools.)

The secondary Schools should be made more efficient, and connected as intimately as possible with the Medical and the Surgical departments of the local Hospitals. They ought also to be withdrawn from the hands of the local authorities, by whom they are now regulated, and placed under the jurisdiction of the University. Students educated at them should be obliged to pass an examination at the close of the second year's studies, previous to being allowed to commence the third.

All legally qualified members of the medical body in France should have the right by law, to teach the medico-chirurgical sciences, and government ought, in Paris and in the principal provincial towns, to place at the disposal of medical practitioners thus wishing to teach any branch of medicine an appropriate locality, and afford them every assistance.—At the same time, lectures thus given are not to interfere with the official instruction given at the faculties and secondary schools, nor to constitute a title towards obtaining University degrees. In a word, they are merely to represent opinions and doctrines.

The authorities are requested to insert in the new medical law a special article, favoring in every possible way clinical teaching by Physicians and Surgeons to Hospitals, both in the metropolis and in the provinces, and giving them the greatest latitude on this score.

The nomination of professors in the faculties of medicine, in the special Schools of Medicine, and in the Veterinary Schools, ought always to take place by concours. (The concours has adversaries in France, and was warmly attacked by them in the Congress, but without success.)

The functions of professors ought to be temporary, and to cease at the age of sixty-five. The professors should then become honorary, and take a part in the board-meetings, deliberations, concours, and administrative labors of the faculties and secondary schools, but without participating in the examinations. They should enjoy their entire salary until the age of seventy, and then retire.

The mechanism adopted for the concours in the faculties ought also to be adopted in the secondary medical schools—that is, one-third of the judges should be taken from among the professors, one-third from the medical academies or societies, and one-third from practising members of the profession.

The existing institution of assistant professors in the faculties, (*aggreges*,) appointed by concours, is approved of, and it is suggested that their salaries should be increased.

The preliminary literary guarantees now demanded of pupils who enter the medical profession, are deemed sufficient, but not too onerous. These guarantees are a bachelor's degree in letters, (arts,) and a bachelor's degree in sciences. They pre-suppose a complete classical and scientific education, of at least ten years' duration.

It is thought expedient to prolong the duration of medical studies from four years to five, to exact an examination at the end of each year, and to oblige all students to serve for at least a year as dressers in some hospital.

The admission and final examinations of doctors should be entrusted to a jury formed partly of professors and regular examiners, and partly of practitioners. The examinations of students should be made even still more practical than they now are. In addition to the five examinations already exacted, a sixth should be added on the subjects treated of in the new professorships of medical philosophy and history.

Such are the views of the congress with reference to that portion of the medical organization of France which relates to teaching and examining pupils, and to receive candidates. The results arrived at with respect to questions relating to the exercise of the medical profession are still more important.

The first question discussed was that of the existence of two orders of medical practitioners. The expediency of having an inferior grade of practitioners was denied, and the institution of officers of health was declared illegitimate and dangerous. This opinion was clearly enounced in the following resolution, which was carried unanimously :—That it is to be hoped that in any future medical law one class of medical practitioners only will be recognised,that of doctors in medicine. That if such a decision be come to, existing officers of health might be allowed, during five years, to offer themselves as candidates for the M. D. degree at any of the faculties.

It was also decided at the same time, that the institution of district medical officers, to take care of the poor, was not necessary and would be an attack on the liberty of the medical body. (This decision of the congress was arrived at in the opposition to the wishes generally expressed in the documents received from the provinces.)

It was proposed, that debts for medical and surgical services should be recoverable by law during a period of five years from the time they are contracted, instead of during one year only, as at present.

The responsibility of a medical practitioner in a medical case cannot be estimated by non-medical persons. In any case, therefore, in which his responsibility is called into question, the penal articles of the code ought only be applied after a decision has been obtained from a medical jury.

In no case ought a medical praciioner be obliged to give evidence against his patients, or to reveal to the authorities, facts which have become known to him in the exercise of his profession.

The exercise of medical practice should be confined strictly to persons qualified by law, and any infringement should be visited by efficacious and energetic punishment. The treatment of sick persons by a non-qualified person ought to be considered penal, even should his prescriptions have been signed by a qualified practitioner.

Councils of discipline should be organized in different parts of the country, and endowed with sufficient power to render their jurisdiction efficacious.

Foreign practitioners ought not be admitted to practice until they have given to government the same guarantees that are demanded of native practitioners. Political refugees, however, should be exempted from all the expenses of examination and admission to the M. D. degree.

It would be inexpedient, unjust and impossible, to limit the number of medical practitioners. The medical profession ought to remain, above all others, a liberal profession.

It was decided, that all advertisements, by means of newspapers, placards, prospectuses, pamphlets, or other modes of publicity, with a view to announce the arrival of a medical practitioner in a locality, his address, a particular means of treatment, or the sale of any medicinal preparation ought to be forbidden, under a severe penalty. That by the word medicinal preparation, should be understood all simple and compound substances or preparations, announced, sold or distributed, as possessing medicinal properties.

That none but "pharmaciens" should be allowed to prepare, to sell, or even to distribute gratuiously, any pharmaceutical preparation. That druggists should be allowed to trade wholesale in drugs, but not to sell them by pharmaceutical weight.

That there should be no exception to the foreign clause, except in favor of persons now trading as "herborists," who might still be allowed to sell non-poisonous and indigenous medicinal plants ; but that in future no one should be allowed to commence business as a "herborist."

That hospitals and other charitable or religious communities should only be allowed to have a pharmacy for their particular use, and should not sell nor distribute out of doors any medicinal substances.

That the existing laws which forbid the sale of secret remedies should be enforced.

That no pharmacien should be allowed to keep two establishments.

That no person should unite in his own person the profession of medical practitioner and that of pharmaçien.

That medical practitioners not residing within a certain reasonable distance of a legally qualified pharmacien, shall be allowed to furnish medicines to their patients, provided the residence of such patients, should be similarly situated in that respect ; but that on a pharmacien settling within the prescribed limits, the privilege should cease.

That a public or private partnership or connection between a medical practitioner and a pharmacien should be forbidden under a severe penalty. That all collusion between medical practitioners and persons foreign to the healing art should likewise be severely forbidden.

That each year the faculties and preparatory schools should choose, by means of the concours, from amongst the pupils who have studied during two years, a certain number to whom scholarships would be given, in order to enable them to continue their studies at the faculties.

That government should establish, in the localities where mineral waters exist, asylums or hospitals for the use of the poor. That government should take proper measures to ensure the admission of pauper patients from rural districts in the hospitals of the department or district. That government should take necessary measures in order to ensure the reception into the hospitals of travelling poor provided with passports of indigence, should they fall ill on their journey. (These passports entitle those to whom they are granted to a small sum per mile.)

That in the rural parishes, as well as in more important parishes, there should be dispensaries established in order to furnish to the poor medicinal and pharmaceutical remedies, the medical officers of which might be entrusted with the registration of births and deaths in small localities.

That the physicians and surgeons to hospitals and other charitable institutions should always form a part, in proper proportion, of the administrative Board.

That each year the physicians, surgeons and pharmaciens of all the hospitals and establishments of charity should be called upon to meet, and to make the competent authorities a report on the imperfections existing in their establishment, and on the ameliorations required. That copies of these reports should be sent to the heads of the administration to which the charity belongs, to the Minister of the Interior, and to the Prefects of the Departments.

That all physicians and surgeons to hospitals should be named by concours, and for a period of fifteen years only, during five years of which they should be assistants, and during the remaining ten, heads; that after the expiration of the fifteen years they should become honorary, but with the power of again contesting the appointment should they think proper. That no hospital physician or surgeon should have more than sixty beds under his care.

That the physicians connected with the mineral watering towns should be named by concours and submitted to a regular gradation in rank. [At each of the French towns where there are mineral waters there is a physician appointed by government to superintend their administration. These appointments are lucrative and at present in the uncontrolled gift of government.]

That the concours should be adopted for the nominative to all appointments to which it can be applied; that when the concours can be applied, certain rules should be followed as much as possible,—the anterior claims of candidates, their previous success in concours, their scientific labors, their previous services in scientific establishments, &c., being taken into consideration.

That the prerogatives and rank of the medical officers of the army should be modified, so as to render their position more in unison with the real importance of the functions which they fulfil.

That no female should exercise the duties of midwife unless she has shown proof of having received a sufficient amount of primary instruction, and of morality of conduct; and unless she has studied this department of medicine during two years at least. The midwives received in the faculties, and in the preparatory schools, or by a medical committee, in towns where there is a midwifery charity affording sufficient means of instruction, ought to undergo two examinations; one on the theory, the other on the practice of midwifery. Midwives should not be allowed to practice any serious obstetric operation, or to attend in cases of disease. The only operations which they ought to be permitted to perform are bleeding and vaccination.

In conclusion, the congress, instituted in a spirit of concord, wished to finish its labors in a spirit of union, and consequently adopted, as a last proposition, the following resolutions :—

That free medical associations should be formed whenever it is possible, with the double view of perfecting science and of organizing provident institutions.

That these associations, formed in the chief town of each arrondisement, should, by delegates, constitute societies in the chief towns of each department, which would lastly, centralise in Paris.

The above proposition embody the results at which the Parisian congress has arrived, after the most searching investigations and discussions, carried on, firstly, in the committees, and secondly, in the public assemblies. All these discussions and deliberations took place publicly. The report of the secretary, briefly embodying the labors of the congress, was read at the final meeting of that body, on the 14th of November, in the presence of M. de Salvandy, the Minister of Public Instruction, who had been requested to attend the final meeting. After the reading of the report, M. de Salvandy rose, and made a most eloquent and emphatic speech, which we consider of importance, from its showing the position which the medical profession occupies in the estimation of the public authorities in France. * * * * * * * *

